# Elements of Natural Philosophy, Being an Experimental Introduction to the Study of the Physical Sciences

**Published:** 1844-04

**Authors:** 


Art. I.—*Elements of Natural Philosophy; being an Experimental Introduction to the Study of the Physical Sciences.* By Golding Bird, A.M. M.D. F.L.S. &c. &c.—Second Edition, revised and enlarged.—*London*, 1844. 12mo, pp. 480.

We have great pleasure in welcoming a new edition of this excellent work, which we strongly recommended to our readers on its first appearance, a few years since, and which now presents still stronger claims upon their attention. Its form has been altered to that of the beautiful series of Manuals now in course of publication by our enterprising publisher Mr. Churchill; but though the size of the book has been thus reduced, the amount of matter it contains has been considerably increased. Three new chapters have been added, embracing Thermotics, or the Science of Heat, (the introduction of which we had suggested in our former notice,) and an account of the Chemical Action of Light, in regard to which so many interesting and important discoveries have been made during the last few years. The chapters on Electrical Decomposition, and part of those on Polarized Light have been rewritten, so as to bring them up to our present more advanced state of knowledge; and many improvements, calculated to elucidate whatever appeared obscure, and to facilitate the labours of the student, have been effected in various parts of the work—not among the least of them being the addition of about eighty additional woodcuts. We do not hesitate to pronounce this little volume the best Manual of Natural Philosophy in our language; and we much doubt if it is equalled—surpassed it can scarcely be—in any other. We trust that the simple and concise form in which the most important truths of physical science are thus presented, will not be inoperative in leading the medical student to gain that degree of acquaintance with them, which is valuable alike as a preparation for his professional pursuits, and as enabling him to take that superior station in intelligent society which he ought to maintain, and which, in these days of general diffusion of scientific information, is not so easily acquired as formerly. Dr. Golding Bird's labours should be not the less acceptable to those who desire to prevent the knowledge which they may have formerly acquired on these subjects, from being forgotten; and who aim at keeping pace, in some degree, with the rapid advance which the physical sciences are at present making.

